# Sequential or Combination Treatments as Rescue Therapies in Immunocompromised Patients with Persistent SARS-CoV-2 Infection in the Omicron Era: A Case Series

**DOI:** 10.3390/antibiotics12091460

**Published:** 2023-09-19

**Authors:** Bianca Maria Longo, Francesco Venuti, Alberto Gaviraghi, Tommaso Lupia, Fabio Antonino Ranzani, Andrea Pepe, Laura Ponzetta, Davide Vita, Tiziano Allice, Vanesa Gregorc, Pio Manlio Mirko Frascione, Francesco Giuseppe De Rosa, Andrea Calcagno, Stefano Bonora

**Affiliations:** 1 Unit of Infectious Diseases, Department of Medical Sciences, University of Turin, “Amedeo di Savoia” Hospital, ASL “Città di Torino”, 10060 Turin, Italyf.venuti@unito.it (F.V.); davide.vita@unito.it (D.V.); francescogiuseppe.derosa@unito.it (F.G.D.R.);; 2 Unit of Infectious Diseases, Cardinal Massaia Hospital, 14100 Asti, Italy; 3 Microbiology and Molecular Biology Laboratory, “Amedeo di Savoia” Hospital, ASL “Città di Torino”, 10060 Turin, Italy; tiziano.allice@aslcittaditorino.it; 4 Unit of Oncology and Haematology, Candiolo Cancer Institute, FPO-IRCCS, 10060 Candiolo, Italy; 5 Department of Medical Sciences, Infectious Diseases, University of Turin, 10126 Turin, Italy

**Keywords:** SARS-CoV-2, persistent infection, antiviral therapy, monoclonal antibodies, HIV/AIDS, immunosuppressive therapy, hematological malignancies

## Abstract

Prolonged SARS-CoV-2 infections are widely described in immunosuppressed patients, but safe and effective treatment strategies are lacking. We aimed to outline our approach to treating persistent COVID-19 in patients with immunosuppression from different causes. In this case series, we retrospectively enrolled all immunosuppressed patients with persistent SARS-CoV-2 infections treated at our centers between March 2022 and February 2023. Patients received different sequential or combination regimens, including antivirals (remdesivir, nirmatrelvir/ritonavir, or molnupiravir) and/or monoclonal antibodies (mAbs) (tixagevimab/cilgavimab or sotrovimab). The main outcome was a complete virological response (negative SARS-CoV-2 RT-PCR on nasopharyngeal swabs) at the end of treatment. Fifteen patients were included as follows: eleven (11/15; 73%) with hematological disease and four (4/15; 27%) with recently diagnosed HIV/AIDS infection. Six patients (6/15; 40%) received a single antiviral course, four patients (4/15; 27%) received an antiviral and mAbs sequentially, and two patients (13%) received three lines of treatment (a sequence of three antivirals or two antivirals and mAbs). A combination of two antivirals or one antiviral plus mAbs was administered in three cases (3/15, 20%). One patient died while still positive for SARS-CoV-2, while fourteen (14/15; 93%) tested negative within 16 days after the end of treatment. The median time to negativization since the last treatment was 2.5 days. Both sequential and combination regimens used in this study demonstrated high efficacy and safety in the high-risk group of immunosuppressed patients.

## 1. Introduction

Persistent severe acute respiratory syndrome coronavirus-2 (SARS-CoV-2) infections have been widely described in specific populations of immunocompromised patients with primary immunodeficiency, onco-hematological malignancies—particularly those receiving B-cell depletion with anti-CD20 antibodies or chimeric antigen receptor T-cell therapies—and patients living with HIV/AIDS (PLWHA) [1,2,3,4,5,6,7].

The risk of sustained viral shedding in immunocompromised hosts has been often reported in recent case series [3,4,5,6,7]. Lee et al. showed an incidence of SARS-CoV-2 RNA detection on nasopharyngeal samples of approximately 13.9% (54/382) beyond day 30 among a cohort of 382 patients diagnosed with hematological malignancies. Furthermore, Dulery et al. reported that patients with hematological malignancies treated with B-cell depleting immunotherapy (i.e., rituximab) experienced prolonged in-hospital stays and increased mortality; in addition, recurrent interstitial pneumonia requiring oxygen supplementation was also observed [8]. Interestingly, persistent infection in immunocompromised hosts is also associated with genomic evolution and the selection of resistance to neutralizing antibodies and antiviral agents [9,10,11]. From medical and public health perspectives, persistent and recurrent SARS-CoV-2 infections in immunocompromised hosts have relevant consequences, and several issues are still unresolved. Firstly, there is a real need for preventive and infection control measures in immunocompromised patients with sustained viral shedding, notably during hospital stays [12]. Secondly, there is a risk of delay in anti-neoplastic or immunosuppressive treatments in SARS-CoV-2-positive immunocompromised hosts [13,14]. Thirdly, safe and effective antimicrobial strategies are required to manage recurrent and persistent immunocompromised carriers.

Even though several antiviral agents and monoclonal antibodies have been introduced to treat SARS-CoV-2 infection or prevent its serious complications, treatment strategies for immunocompromised patients with persistent infection lack data from clinical trials and are limited to heterogenous case reports and series [15,16,17].

Therefore, the aim of the case series was to describe the outcomes of different rescue therapies for persistent SARS-CoV-2 infection in immunocompromised subjects treated in our clinical setting during the last two seasons (2021–2022 and 2022–2023) of the COVID-19 pandemic.

## 2. Results

Fifteen immunocompromised patients with persistent SARS-CoV-2 were included. Our analysis was carried out during the era of the SARS-CoV-2 Omicron variant, ranging from the original strain B.1.1.592 to the current sub-variant XXB. Based on national data, BA.4, BA.5, and BQ.1 were the predominant subvariants at the time of our analysis. Among these patients, 9 (60%) were males, and the median age was 65 (IQR 58.3–73) (Table 1).

In accordance with the literature [1,2,3,4,5,6,7], the main risk factor associated with persistent SARS-CoV-2 infections among our patients was an onco-hematological disease, which was present in 11 participants (73%) and patients (27%) recently diagnosed with HIV/AIDS infection. Among other comorbidities reported in these immunocompromised hosts, type 2 diabetes mellitus (27%) and cardiovascular disorders were the most represented (40%).

Among the onco-hematological patients, 8 (73%) were diagnosed with non-Hodgkin lymphoma (NHL), while the remaining cases included Hodgkin lymphoma (HL; 9%), chronic lymphatic leukemia (CLL; 9%), and hypogammaglobulinemia (9%). Moreover, 73% of these patients underwent immunomodulatory therapy during COVID-19 treatment, and 64% had received an anti-CD20-based regimen in the past six months. In addition, one patient had undergone hematopoietic stem cell transplantation (HSCT) and one had received CAR-T cell therapy.

All participants newly discovered to live with HIV presented a low CD4+ count (median number of 21 cells/µL) and concomitant opportunistic infections (i.e., *Pneumocystis jirovecii* pneumonia and disseminated cytomegalovirus infection). Three were treatment-naïve individuals at the onset of COVID-19 and were started on antiretroviral treatment while still positive for SARS-CoV-2 (median time from first positive swab of 151 days). One patient had started antiretroviral treatment 63 days before the first positive swab but presented a low CD4+ count at COVID-19 diagnosis (CD4+ 20 cells/µL). Regardless of timing, all patients with HIV began an antiretroviral regimen, including two nucleotide reverse transcriptase inhibitors (NRTIs) and one integrase strand transfer inhibitor (INSTI).

All participants were vaccinated with two or more doses of anti-SARS-CoV-2 (except for one patient who had been vaccinated with a single dose only). The median time between the last dose of the vaccine and the diagnosis of the SARS-CoV-2 infection was 408 days. In addition, four patients (26%) had received tixagevimab/cilgavimab prophylaxis before the SARS-CoV-2 infection.

Furthermore, all patients were symptomatic for SARS-CoV-2 infection (53% with mild symptoms, 33% with moderate symptoms, and 13% with severe symptoms). Pneumonia was detected on CT scans in nine patients (60%). According to the severity of symptoms, 11 out of 15 cases (73%) needed hospitalization, 7 of which (47%) required oxygen support and concomitant courses of steroids (4 with nasal cannula and 3 with non-invasive ventilation (NIV), 1 of whom had concomitant *Pneumocystis jirovecii* pneumonia and required admission to the ICU).

Eleven patients (73%) received early treatment with antiviral or monoclonal antibodies within the first 10 days after the detection of SARS-CoV-2. As early treatment, the most commonly used regimen (55% of cases) was a five-day nirmatrelvir/ritonavir 300 mg/100 mg q12h. None of these patients achieved negativization after early therapy administration.

Regardless of whether early therapy was administered, all the patients reported developing persistent SARS-CoV-2 infection and required antiviral and/or monoclonal therapies outside the conventional regimens and timings. The median time from the first positive swab to the rescue therapy for persistent SARS-CoV-2 infection was 28 days (IQR 24.5–44.5), with a median of two drugs per patient. Only 27% of cases received more than two courses of therapy.

**Figure 1:**
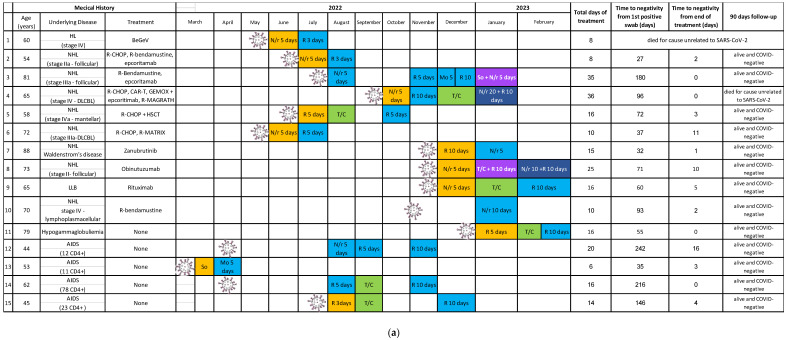

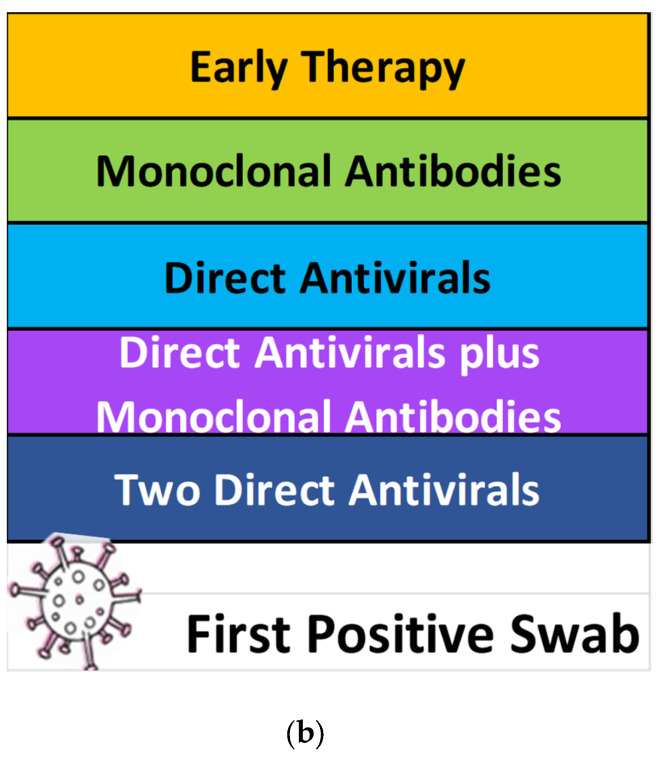
(**a**) Timing, regimens, and outcomes of treatments administered to the patients during the study period. Abbreviations: AIDS: acquired immunodeficiency syndrome; RDV: remdesivir; T/C: tixagevimab/cilgavimab; COVID: coronavirus disease; Mo: molnupiravir; So: sotrovimab; N/r: nirmatrelvir/ritonavir; NHL: non-Hodgkin lymphoma; R: rituximab; R-CHOP: cyclophosphamide, doxorubicin, prednisone, rituximab, and vincristine; GEMOX: gemcitabine hydrochloride and oxaliplatin; LLB: lymphocytic leukemia-B; HL: Hodgkin lymphoma; SARS-CoV-2: severe acute respiratory syndrome coronavirus-2. DLCBL: diffuse large B cell lymphoma, CD4+: explanation not necessary, BeGeV: bendamustine, gemcitabine, and vinorelbine, CAR-T: chimeric antigen receptor-T cell therapy, R-MAGRATH: rituximab, cyclophosphamide, vincristine, doxorubicin-methatrexate, ifosfamide, etoposide, cytarabine, HSCT: Hematopoietic stem cell transplantation, R-MATRIX: rituximab, methotrexate, cytarabine, and thiotepa. (**b**) Legend for subfigure (**a**).

As rescue therapy, six patients (40%) received a single antiviral course (Figure 1a) as follows: remdesivir for three or five days; nirmatrelvir/r for five or ten days; and molnupiravir for five days. 

All of them had mild symptoms with no need for oxygen therapy, except for one case that presented with moderate disease and a need for nasal cannula support.

Four patients (27%) received sequentially an antiviral treatment (i.e., remdesivir) and monoclonal antibodies (i.e., tixagevimab/cilgavimab). Persistent positivity after administration of the first drug guided the decision to administer a second molecule with a different mechanism of action. Again, the duration of remdesivir varied from 5 to 10 days, depending on the severity of the disease.

For the same reason, two patients (13%) received three lines of treatment to obtain viral clearance and clinical recovery. In the first case, a sequence of three antivirals was selected (nirmatrelvir/r for five days, remdesivir for five days, and remdesivir for ten days), while in the second case, two antivirals and monoclonal antibodies were used sequentially (remdesivir for five days and tixagevimab/cilgavimab and remdesivir for ten days).

Moreover, due to persistent positivity, a combination therapy was administered to three patients (20%) who failed to achieve negativization with previous sequential treatments. The regimens selected were oral nirmatrelvir/ritonavir for 10 days plus IV sotrovimab single dose, nirmatrelvir/r for 20 days plus remdesivir for 10 days, and nirmatrelvir/r for 10 days plus remdesivir for 10 days. The use of the first two combinations led to the achievement of negativization by the end of the course of treatment; differently, as for the last regimen, remdesivir was stopped after 7 days due to hepatotoxicity with full recovery after cessation of the drug, and negativization was obtained 10 days later.

One patient died while still positive for SARS-CoV-2 from a cause unrelated to the infection. All the other patients under review (14/15) achieved negativization within 16 days from the completion of treatment (33% after a single-treatment course, 27% after two courses, 27% after three, and 7% after four courses or more). Overall, the median time to negativization since the first positive swab was 71 days (IQR 41.5–133.5), while the median time to negativization since the last treatment was 2.5 days (IQR 0.25–4.75).

At the 90-day follow-up, two patients had died because of causes unrelated to COVID-19 (one was negative for SARS-CoV-2, and one was still positive); 13 patients were alive, asymptomatic, and negative for SARS-CoV-2.

## 3. Discussion

In this case series, we describe the clinical–virological characteristics and outcomes in immunocompromised patients with persistent SARS-CoV-2 who underwent rescue therapies with sequential or combination of licensed treatments for SARS-CoV-2.

Recently, Mikulska et al. [7] presented one of the larger cohorts of immunocompromised patients with persistent SARS-CoV-2 who received antiviral/antibody combination therapy. The authors reported a median age similar to our retrospective study (70 vs. 65), with an equally higher prevalence of male patients [7]. In Italy, from the beginning of the pandemic in March 2020, we faced approximately 26,000,000 COVID-19 cases, with a mortality rate of 0.7% [18]. Unlike our cohort and Mikulska et al., most Italian cases occurred in younger patients (median age 45 vs. 65), of which 53.5% involved females [18]. In a large study that included patients from 16 countries, Yanez et al. found that the COVID-19 mortality rates were significantly higher in individuals older than 65 years of age and in males compared to younger patients and females, respectively. Along with the known state of immunosuppression, we collected a cohort of patients at high risk of morbidity and mortality due to SARS-CoV-2 infection [19].

Moreover, all our study participants presented with typical SARS-CoV-2 infection symptoms in a range from mild to severe, and pneumonia was detected on CT scans in 60% of the patients. This result is comparable with larger studies available in the literature [7,20,21,22,23].

Our population was composed mainly of onco-hematological patients, in accordance with previously reported experiences [7,20,21,22,23]. Interestingly, to our knowledge, this is the first report including up to four PLWHA with persistent SARS-CoV-2 infection treated with sequential or combined antiviral/antibody therapies. Reports from the literature on persistent SARS-CoV-2 infections in PLWHA are rapidly increasing, despite a lack of experience and data regarding therapies for their management. Furthermore, different authors have reported a high risk of the formation of mutant SARS-CoV-2 strains in immunocompromised hosts, including those with advanced HIV disease [10,24], increasing the risk of immune escape of vaccines and SARS-CoV-2 variants, with implications for vaccine breakthroughs and reinfections [10,24]. Further, PLWHA described in this study was characterized by a profound immune-virological deterioration with a median CD4+ nadir of 21 cells/µL, and an antiretroviral combination therapy started close to SARS-CoV-2 rescue therapies.

All patients within our population received a complete course of SARS-CoV-2 vaccination, with the exception of one patient vaccinated with a single dose. Recently, data from 20,431 COVID-19 patients were published by Riemersma et al. When adjusting for vaccine product or sex and comparing samples from vaccinated and unvaccinated people, no significant effect of vaccine status alone on viral shedding was observed [25]. These observations could be explained by the immune-escape risk of the latter variants from vaccine coverage, especially in immunocompromised hosts.

In our study, four patients (26%) had received tixagevimab/cilgavimab prophylaxis before a SARS-CoV-2 infection. This result is comparable with the one obtained by Mikulska et al. No other large study involving onco-hematological patients who received tixagevimab/cilgavimab prior to a SARS-CoV-2 infection is present in the literature. The breakthrough infection rate by SARS-CoV-2 after prophylaxes with mAbs like tixagevimab/cilgavimab in immunocompromised patients should be investigated with further studies.

Most of the patients reported (73%) underwent early antiviral or antibody therapy after the first COVID-19 diagnosis. There is a lack of information on current medication efficacy in slowing disease development in immunocompromised people newly diagnosed with COVID-19. However, various antiviral medications against SARS-CoV-2 have been related to accelerated viral clearance and better COVID-19 outcomes among immunocompromised groups. Remdesivir, for instance, can hasten viral clearance in B-cell- and antibody-deficient hosts with recurrent or chronic COVID-19 [26]. In adults with symptoms and at least one risk factor for severe COVID-19, the newer oral antiviral combination of nirmatrelvir/ritonavir has been demonstrated to reduce the rate of progression to hospitalization/death (0.77% vs. 7.00%) [27]. Notably, Hammonds et al.’s seminal study also involved populations at risk, such as patients with HIV, cancer, and iatrogenic immunosuppression.

In our study, the following four different therapy schemes were administered to patients with persistent infections to achieve viral clearance: single antiviral, sequence of antivirals, sequence of antivirals and monoclonal antibodies, and double combination therapy. Most of the patients considered (40%) received a single antiviral course of remdesivir, nirmatrelvir/ritonavir, or molnupiravir. The choice of the drug and its duration were guided by patients’ clinical conditions and immunological status, including treatment setting, comorbidities, and possible drug–drug interactions.

Various treatment schemes have been analyzed in the literature. Brown et al. [26] surveyed immunologists in the United Kingdom to collect data on immunosuppressed adults with prolonged or relapsing COVID-19. Clinical data from 31 patients with primary or secondary immunodeficiencies were collected. Of the 20 patients in whom the virus was cleared, 13 received a combination of remdesivir and an antibody-based therapy (either mAbs or convalescent plasma), and 7 received remdesivir monotherapy, 2 of which lasted >10 days. Of the eleven patients who failed to reach viral clearance, one received combination therapy (remdesivir plus convalescent plasma), seven received remdesivir monotherapy, and three did not receive any antiviral treatment.

In our retrospective study, 27% of the patients received a combination of antiviral treatment (i.e., remdesivir) and monoclonal antibodies (i.e., tixagevimab/cilgavimab). Brown and colleagues [26] concluded that combination therapy with antiviral and antibody-based therapeutics was better associated with viral clearance than monotherapy with an antiviral (92.8% vs. 50%). The odds ratio of clearing infection through the administration of combination therapy versus remdesivir monotherapy was 23.1 (95% CI 5 1.3-424.9 [P 0.035]). Moreover, Wada et al. [28] described ten patients with hematological malignancies and persistent COVID-19 who were treated with a combination of antiviral and monoclonal antibodies. Remdesivir was chosen as the initial treatment option but was eventually switched to nirmatrelvir/ritonavir in four patients and to molnupiravir in one patient due to the lack of reduction in viral load or CT values. All patients obtained viral clearance, and none suffered a relapse of the viral infection.

Two patients enrolled in our study received three lines of treatment, including at least two antivirals, as reported in Figure 1. A combination of remdesivir and nirmatrelvir/ritonavir has been described as a successful treatment option in two case reports of immunocompromised patients with hematological malignancies [29,30]. The potential synergism of antivirals targeting different mechanisms of viral replication, such as RNA polymerase for remdesivir and protease for nirmatrelvir/r, has been demonstrated in vitro [31]. A longer course of therapy may also be necessary for these patients, considering the eventuality of a rebound of viral shedding and symptoms described after standard courses of nirmatrelvir/ritonavir, possibly due to inadequate drug exposure [32,33]. The enhanced efficacy of combined antiviral therapy was confirmed by our cohort, as it was used in two patients who had failed previous treatment courses of antiviral monotherapy or antibodies.

In our study, severe adverse events occurred in the case of one patient treated with remdesivir and nirmatrelvir/ritonavir due to suspected hepatotoxicity. However, this was resolved after remdesivir discontinuation.

The main findings were the high efficacy of both sequential (91%) and combination therapies (100%) in terms of virological response and the high safety, with very low risk of moderate–severe adverse events (1 of 15 patients). In the work by Mikulska et al. [7], 22 cases of severely immunocompromised patients with prolonged/relapsed COVID-19 received combination treatment with two antivirals associated with monoclonal antibodies in 18 of the 22 cases. The most frequent antiviral combination was remdesivir plus nirmatrelvir/ritonavir. Early virological response (negative SARS-CoV-2 swab) at day 14 was achieved in 15/20 evaluated patients (75%), while 30-day virological and clinical response was achieved in 16/22 patients (73%). Interestingly, the rates of virological response at day 14 and virological and clinical response at day 30 were significantly higher in the case of the combination treatment including mAbs (*p* = 0.032 and *p* = 0.046, respectively).

Our case series has various limitations. First, it is a case series, and due to the nature of the manuscript, statistical tests yielding *p* values or confidence intervals are not generally used by a large part of the authors [34]. Secondly, precise viral genotyping is lacking, and possible reinfections from different viral variants rather than prolonged courses of the same virus cannot be excluded. However, the clinical course of infections in our patients is consistent with prolonged infections. Third, we have described a small group, and there was no control group. Fourth, we considered patients with different types of immune suppression who had been infected with SARS-CoV-2 for different lengths of time before receiving combination therapy. Finally, it is not easily assessable if negativization was due to the natural course of infection rather than to the effect of our treatments or to an interplay between these two factors. This is especially true for patients with several lines of treatment and a longer persistence of infection. Nevertheless, the short mean time to negativization since the end of treatment represents an encouraging result.

## 4. Materials and Methods

This case series included immunocompromised patients who received antiviral and/or antibody combination therapies for persistently symptomatic SARS-CoV-2 infections. Patients were enrolled between 1 March 2022 and 1 February 2023 at the Clinic of Infectious Diseases of the University of Turin, Amedeo di Savoia Hospital (Turin, Italy).

A diagnosis of SARS-CoV-2 infection was established by real-time reverse-transcriptase polymerase chain reaction (RT-PCR) testing and/or LumiraDx antigenic tests performed on nasal swabs. According to disease severity, tests were performed weekly or every 72 h until the end of follow-up or negativization.

Persistent SARS-CoV-2 infection was defined in accordance with confirmation from RT-PCR testing and/or LumiraDx antigenic tests performed on nasal swabs after 21 days from the first SARS-CoV-2 positive test. Because of persistent infection, worsened by compromised immune status, antivirals and monoclonal antibodies beyond the conventional timings established by current international guidelines were administered. Off-label protocols received individual approval from the dedicated hospital committee. All patients signed informed consent for treatment and data collection. Further, all patients underwent routine weekly follow-up testing until a negative RT-PCR or antigenic result was obtained. The minimum follow-up period after completion of treatment was 90 days.

The antiviral molecules used in the study were remdesivir (200 mg IV as a loading dose, followed by 100 mg for a total duration of 3, 5, or 10 days), nirmatrelvir/ritonavir (300/100 mg q12h po for 5–20 days), and molnupiravir (800 mg q12h po for 5 days), alone or in combination. The antibodies used were tixagevimab/cilgavimab (300/300 mg IM single dose) or sotrovimab (500 mg IV single dose), alone or sequential. Therapies were administered in sequential regimens or as combination treatments. The choice of the regimen and its duration were guided by the severity of the disease, renal and liver function, and the evolution of viral variants over time. Our analysis was carried out during the era of the SARS-CoV-2 Omicron variant, ranging from the original strain B.1.1.592 to the current sub-variant XXB. Based on national data, BA.4, BA.5, and BQ.1 were the predominant subvariants at the time of our analysis [35]. In line with this, in two of our cases, a 69–70 spike deletion, which is highly suggestive for BA.4 or BA.5 sub-variants, was detected on molecular examination. All the proposed treatment regimens were discussed collegially before initiation and re-evaluated weekly. Moreover, additional therapies (e.g., corticosteroids, heparin, or antibiotics) were administered in accordance with disease severity and clinical needs.

A complete virological response was defined as negative SARS-CoV-2 RT-PCR (with or without a negative coupled SARS-CoV-2 antigenic test) in nasopharyngeal swabs at the end of treatment.

Categorical variables were described using absolute numbers and percentages, while continuous variables were presented using the median and interquartile range (IQR). All the analyses were performed using the statistical package for social sciences (SPSS for Windows, version 21.0; SPSS Inc., Chicago, IL, USA).

## 5. Conclusions

Immunocompromised patients are at high risk of persistent and symptomatic COVID-19 despite extensive vaccination. The best antiviral/monoclonal antibodies for clearing SARS-CoV-2 are unclear, and our case series provides potential combinations and sequential regimens for this high-risk group of patients.

## Figures and Tables

**Table 1:** Clinical, laboratory, and treatment characteristics of the population involved in the study.

Characteristics	N (%)	IQR
Male sex	9 (60)	
Age (median)	65	(58–73)
Underlying diseases	N (%)	
Onco-hematological disease	11 (73)	
Non-Hodgkin lymphoma	8 (72)	
Hodgkin lymphoma	1 (9)	
Chronic lymphocytic leukemia	1 (9)	
Hypogammaglobulinemia	1 (9)	
HSCT	1 (9)	
Other causes of immunocompromise		
Ongoing immunomodulant therapies (other than anti-CD20 biologics)	8 (73)	
Anti-CD20 treatment within 6 months	7 (64)	
HIV/AIDS patients and characteristics	4 (27)	
nadir CD4+T-cell count, median	23	(12–78)
Treatment-naïve at COVID-19 onset	3 (75)	
Concomitant opportunistic infections	4 (100)	
Comorbidities of immunocompromised population	N (%)	
Cardiovascular diseases	6 (40)	
Type 2 diabetes mellitus	4 (27)	
BMI > 30	2 (13)	
Chronic respiratory disease	2 (13)	
SARS-CoV-2 prophylaxis	N (%)	
Vaccinated for SARS-CoV-2	15 (100)	
No. of doses of vaccine, median	3	(1–5)
Time to diagnosis, days, from last dose of vaccine, median	408	(337–447)
Prophylactic monoclonal antibodies	4 (27)	
Clinical parameters	N (%)	
Symptomatic for SARS-CoV-2	15 (100)	
Mild symptoms	8 (53)	
Moderate symptoms	5 (33)	
Severe symptoms	2 (13)	
Pneumonia	9 (60)	
Hospitalization	11 (73)	
Oxygen therapy	7 (47)	
Nasal cannula	4 (57)	
NIV	3 (29)	
ICU admission	1 (33)	
Adjuvant treatment with steroids	7 (47)	
Previous SARS-CoV-2 therapies	N (%)	
Previous early therapy for the same SARS-CoV-2 infection episode	11 (73)	
Remdesivir for 3 days	1 (9)	
Remdesivir for 5 days	2 (18)	
Remdesivir for 10 days	1 (9)	
Molnupiravir 5 days	0 (0)	
Nirmatrelvir/ritonavir for 5 days	6 (55)	
Tixagevimab/cilgavimab	0 (0)	
Sotrovimab	1 (9)	

Abbreviations: N: number; IQR: interquartile range; NIV: non-invasive ventilation; ICU: intensive care unit; BMI: body mass index; COVID: coronavirus disease; SARS-CoV-2: severe acute respiratory syndrome coronavirus; HIV/AIDS: human immunodeficiency virus/acquired immunodeficiency syndrome; HSCT: hematopoietic stem cell transplantation.

## Data Availability

Data will be available upon appropriate request to the corresponding author.

## References

[1] Aydillo T., Gonzalez-Reiche A.S., Aslam S., van de Guchte A., Khan Z., Obla A., Dutta J., van Bakel H., Aberg J., García-Sastre A. (2020). Shedding of viable SARS-CoV-2 after immunosuppressive therapy for cancer. N. Engl. J. Med..

[2] Choi B., Choudhary M.C., Regan J., Sparks J.A., Padera R.F., Qiu X., Solomon I.H., Kuo H.H., Boucau J., Bowman K. (2020). Persistence and evolution of SARS-CoV-2 in an immunocompromised host. N. Engl. J. Med..

[3] Lee C.Y., Shah M.K., Hoyos D., Solovyov A., Douglas M., Taur Y., Maslak P., Babady N.E., Greenbaum B., Kamboj M. (2022). Prolonged SARS-CoV-2 infection in patients with lymphoid malignancies. Cancer Discov..

[4] Sepulcri C., Dentone C., Mikulska M., Bruzzone B., Lai A., Fenoglio D., Bozzano F., Bergna A., Parodi A., Altosole T. (2021). The longest persistence of viable SARS-CoV-2 with recurrence of viremia and relapsing symptomatic COVID-19 in an immunocompromised patient—A case study. Open Forum Infect. Dis..

[5] Westblade L.F., Brar G., Pinheiro L.C., Paidoussis D., Rajan M., Martin P., Goyal P., Sepulveda J.L., Zhang L., George G. (2020). SARS-CoV-2 viral load predicts mortality in patients with and without cancer who are hospitalized with COVID-19. Cancer Cell.

[6] Belkin A., Leibowitz A., Shargian L., Yahav D. (2023). The unique presentation of SARS-CoV-2 infection in patients with B-cell depletion: Definition of “persistent inflammatory sero-negative COVID”. Clin. Microbiol. Infect..

[7] Mikulska M., Sepulcri C., Dentone C., Magne F., Balletto E., Baldi F., Labate L., Russo C., Mirabella M., Magnasco L. (2023). Triple combination therapy with two antivirals and monoclonal antibodies for persistent or relapsed SARS-CoV-2 infection in immunocompromised patients. Clin. Infect. Dis..

[8] Duléry R., Lamure S., Delord M., Di Blasi R., Chauchet A., Hueso T., Rossi C., Drenou B., Deau Fischer B., Soussain C. (2021). Prolonged in-hospital stay and higher mortality after COVID-19 among patients with non-Hodgkin lymphoma treated with B-cell depleting immunotherapy. Am. J. Hematol..

[9] Gandhi S., Klein J., Robertson A., Peña-Hernández M.A., Lin M.J., Roychoudhury P., Lu P., Fournier J., Ferguson D., Mohamed Bakhash S.A. (2022). De novo emergence of a remdesivir resistance mutation during treatment of persistent SARS-CoV-2 infection in an immunocompromised patient: A case report. Nat. Commun..

[10] Cele S., Karim F., Lustig G., San J.E., Hermanus T., Tegally H., Snyman J., Moyo-Gwete T., Wilkinson E., Bernstein M. (2022). SARS-CoV-2 prolonged infection during advanced HIV disease evolves extensive immune escape. Cell Host Microbe.

[11] Hogan J.I., Duerr R., Dimartino D., Marier C., Hochman S.E., Mehta S., Wang G., Heguy A. (2023). Remdesivir Resistance in Transplant Recipients with Persistent Coronavirus Disease 2019. Clin. Infect. Dis..

[12] Owusu D., Pomeroy M.A., Lewis N.M., Wadhwa A., Yousaf A.R., Whitaker B., Dietrich E., Hall A.J., Chu V., Thornburg N. (2021). Persistent SARS-CoV-2 RNA Shedding without Evidence of Infectiousness: A Cohort Study of Individuals with COVID-19. J. Infect. Dis..

[13] Corey L., Beyrer C., Cohen M.S., Michael N.L., Bedford T., Rolland M. (2021). SARS-CoV-2 Variants in Patients with Immunosuppression. N. Engl. J. Med..

[14] Kareff S.A., Khan A., Barreto-Coelho P., Iyer S.G., Pico B., Stanchina M., Dutcher G., Monteiro de Oliveira Novaes J., Nallagangula A., Lopes G. (2022). Prevalence and Outcomes of COVID-19 among Hematology/Oncology Patients and Providers of a Community-Facing Health System during the B1.1.529 (“Omicron”) SARS-CoV-2 Variant Wave. Cancers.

[15] Cattel L., Giordano S., Traina S., Lupia T., Corcione S., Angelone L., La Valle G., De Rosa F.G., Cattel F. (2022). Vaccine development and technology for SARS-CoV-2: Current insight. J. Med. Virol..

[16] Mornese Pinna S., Lupia T., Scabini S., Vita D., De Benedetto I., Gaviraghi A., Colasanto I., Varese A., Cattel F., De Rosa F.G. (2021). Monoclonal antibodies for the treatment of COVID-19 patients: An umbrella to overcome the storm?. Int. Immunopharmacol..

[17] Bassetti M., Corcione S., Dettori S., Lombardi A., Lupia T., Vena A., De Rosa F.G., Gori A., Giacobbe D.R. (2021). Antiviral treatment selection for SARS-CoV-2 pneumonia. Expert Rev. Respir. Med..

[18] https://www.epicentro.iss.it/coronavirus/sars-cov-2-dashboard.

[19] Yanez N.D., Weiss N.S., Romand J.A., Treggiari M.M. (2020). COVID-19 mortality risk for older men and women. BMC Public Health.

[20] Martínez-Barranco P., García-Roa M., Trelles-Martínez R., Arribalzaga K., Velasco M., Guijarro C., Marcos J., Campelo C., Acedo-Sanz J.M., Villalón L. (2022). Management of Persistent SARS-CoV-2 Infection in Patients with Follicular Lymphoma. Acta Haematol..

[21] Hettle D., Hutchings S., Muir P., Moran E. (2022). COVID-19 Genomics UK (COG-UK) consortium. Persistent SARS-CoV-2 infection in immunocompromised patients facilitates rapid viral evolution: Retrospective cohort study and literature review. Clin. Infect. Pract..

[22] Blennow O., Vesterbacka J., Tovatt T., Nowak P. (2023). Successful combination treatment for persistent SARS-CoV-2 infection. Clin. Infect. Dis..

[23] Marangoni D., Antonello R.M., Coppi M., Palazzo M., Nassi L., Streva N., Povolo L., Malentacchi F., Zammarchi L., Rossolini G.M. (2023). Combination regimen of nirmatrelvir/ritonavir and molnupiravir for the treatment of persistent SARS-CoV-2 infection: A case report and a scoping review of the literature. Int. J. Infect. Dis. IJID.

[24] Maponga T.G., Jeffries M., Tegally H., Sutherland A., Wilkinson E., Lessells R.J., Msomi N., van Zyl G., de Oliveira T., Preiser W. (2023). Persistent Severe Acute Respiratory Syndrome Coronavirus 2 Infection with accumulation of mutations in a patient with poorly controlled Human Immunodeficiency Virus infection. Clin. Infect. Dis..

[25] Riemersma K.K., Haddock L.A., Wilson N.A., Minor N., Eickhoff J., Grogan B.E., Kita-Yarbro A., Halfmann P.J., Segaloff H.E., Kocharian A. (2022). Shedding of infectious SARS-CoV-2 despite vaccination. PLoS Pathog..

[26] Brown L.K., Moran E., Goodman A., Baxendale H., Bermingham W., Buckland M., AbdulKhaliq I., Jarvis H., Hunter M., Karanam S. (2022). Treatment of chronic or relapsing COVID-19 in immunodeficiency. J. Allergy Clin. Immunol..

[27] Hammond J., Leister-Tebbe H., Gardner A., Abreu P., Bao W., Wisemandle W., Baniecki M., Hendrick V.M., Damle B., Simón-Campos A. (2022). Oral nirmatrelvir for high-risk, nonhospitalized adults with COVID-19. N. Engl. J. Med..

[28] Wada D., Nakamori Y., Maruyama S., Shimazu H., Saito F., Yoshiya K., Kuwagata Y. (2022). Novel treatment combining antiviral and neutralizing antibody-based therapies with monitoring of spike-specific antibody and viral load for immunocompromised patients with persistent COVID-19 infection. Exp. Hematol. Oncol..

[29] Ford E.S., Simmons W., Karmarkar E.N., Yoke L.H., Braimah A.B., Orozco J.J., Ghiuzeli C.M., Barnhill S., Sack C.L., Benditt J.O. (2023). Successful Treatment of Prolonged, Severe Coronavirus Disease 2019 Lower Respiratory Tract Disease in a B cell Acute Lymphoblastic Leukemia Patient with an Extended Course of Remdesivir and Nirmatrelvir/Ritonavir. Clin. Infect. Dis..

[30] Trottier C.A., Wong B., Kohli R., Boomsma C., Magro F., Kher S., Anderlind C., Golan Y. (2023). Dual Antiviral Therapy for Persistent Coronavirus Disease 2019 and Associated Organizing Pneumonia in an Immunocompromised Host. Clin. Infect. Dis..

[31] Schultz D.C., Johnson R.M., Ayyanathan K., Miller J., Whig K., Kamalia B., Dittmar M., Weston S., Hammond H.L., Dillen C. (2022). Pyrimidine inhibitors synergize with nucleoside analogues to block SARS-CoV-2. Nature.

[32] Carlin A.F., Clark A.E., Chaillon A., Garretson A.F., Bray W., Porrachia M., Santos A.T., Rana T.M., Smith D.M. (2023). Virologic and immunologic characterization of Coronavirus Disease 2019 recrudescence after nirmatrelvir/ritonavir treatment. Clin. Infect. Dis..

[33] Boucau J., Uddin R., Marino C., Regan J., Flynn J.P., Choudhary M.C., Chen G., Stuckwisch A.M., Mathews J., Liew M.Y. (2023). Characterization of virologic rebound following nirmatrelvir-ritonavir treatment for Coronavirus Disease 2019 (COVID-19). Clin. Infect. Dis..

[34] Carey T.S., Boden S.D. (2003). A critical guide to case series reports. Spine.

[35] Data on SARS-CoV-2 variants in the EU/EEA. https://www.ecdc.europa.eu/en/publications-data/data-virus-variants-covid-19-eueea.

